# Family meals on prescription as treatment for childhood obesity—a randomized controlled trial

**DOI:** 10.1007/s00431-024-05744-8

**Published:** 2024-09-09

**Authors:** Terese Torstensson, Anna Bohlin, Gerd Almqvist-Tangen, Josefine Roswall, Jenny M. Kindblom, Lovisa Sjogren

**Affiliations:** 1 Department of Pediatrics, Institute of Clinical Sciences at the Sahlgrenska Academy at University of Gothenburg, Gothenburg, Sweden; 2 Department of Pediatrics, Hallands Hospital Halmstad, Halmstad, Sweden; 3 Institute of Medicine, Sahlgrenska Academy at University of Gothenburg, Gothenburg, Sweden; 4 Department of Drug Treatment, Sahlgrenska University Hospital, Region Västra Götaland, Gothenburg, Sweden; 5 Department of Pediatrics, Region Västra Götaland, Sahlgrenska University Hospital, Gothenburg, Sweden

**Keywords:** Childhood obesity, Dietary intervention, Body mass index, Family meals

## Abstract

The aim with the present study was to evaluate the effects and tolerability of Family Meals on Prescription, a 3-month intensive dietary intervention with a participatory approach on body mass index (BMI) and metabolic health in children living with obesity. In this prospective randomized controlled trial, children aged 5–15 years were included from the Pediatric Obesity outpatient Clinics in Halland, Sweden. Participants were randomly assigned to receive lifestyle treatment with or without Family Meals on Prescription (FMP) consisting of a subsidized prepacked grocery bag including recipes and provisions for five Family Meals per week for 3 months. The primary endpoint was changed in BMIz after 3, 12 and 18–24 months and secondary endpoints included to assess tolerability of FMP and effects on metabolic biomarker and frequency of shared meals. Eighty-nine children (51.7% female) entered the study, 54 patients in the intervention group and 35 in the control group. There were no significant differences between the groups concerning gender, age or level of obesity at baseline. The Family Meal on Prescription intervention combined with lifestyle treatment led to a significantly greater reduction in BMIz than lifestyle treatment alone after the 3-month long intervention (− 0.17 vs + 0.01, *p* < 0.01); however, this difference was not sustained throughout the study period, and in fact, the control group had a greater reduction in BMIz after 18–24 months.

A subsidized prepacked grocery bag may be a novel, well-tolerated and effective tool in the treatment of childhood obesity. The fact that the BMIz reduction shown at the end of the intervention did not persist over time emphasized the need of long-term treatment. Registered at clinicaltrals.gov 27 Nov 2020, retrospectively registered: clinicaltrials.gov number 19002468. https://clinicaltrials.gov/study/NCT05225350

**Table:** 

**What is Known:** *• Swedish data shows that lifestyle treatment alone is not sufficient for many families undergoing treatment for childhood obesity.* *• Regular family meals and mealtime routines have been shown to be important for nutritional health and dietary patterns in children and adolescents.*	
**What is New:** *• This intervention with a participatory approach involving prepacked family meals was well tolerated and led to a significant reduction in BMIz during the intervention.* *• That fact that these results were not sustained over time indicates a need to evaluate longer interventions, and that childhood obesity is a chronic and complex disease which requires long-time treatments.*

**Supplementary Information:**

The online version contains supplementary material available at 10.1007/s00431-024-05744-8.

## Introduction

Obesity in children and adolescents is a global health challenge. The prevalence of childhood overweight and obesity in Sweden has been reported to be 20–25% and 5%, respectively, making childhood obesity one of the most common chronic conditions [1–3].

The aim of obesity treatment in children is to achieve and maintain modifications in lifestyle as well as to reduce excess weight and BMI z-score (BMIz) [4, 5]. A reduction larger than 0.25 BMIz has been shown to be associated with a reduced prevalence of metabolic syndrome and clinically significant improvements in the metabolic syndrome components [6, 7].

Lifestyle treatment is focused on altering dietary intake, increasing physical activity as well as supporting behavioural changes [4, 5]. Evidence indicates that dietary interventions for children with obesity should focus on both quantity and quality of food, with the aim of lowering the energy intake, have a duration of at least three months and be intensive in character to be effective [8]. However, there is a scarcity of randomized controlled trials evaluating intensive dietary interventions with a participatory and practical approach in the home setting with a long follow-up period indicating a need for further studies [9]. Family-based behavioural treatment has been reported to improve the weight status of children with obesity both in the short and long term [4, 10–12]. Further, there is evidence that regular shared family meals are associated with improved nutritional health and healthy dietary patterns in children and adolescents [13–15]. Of note, increasing numbers of studies investigating the effect of lifestyle interventions delivered in the home setting show promising results [16].

Lifestyle treatment is the foundation of all paediatric obesity treatment in Sweden. The accessibility to care widely differs between Swedish regions. The region of the present study offers all children with obesity lifestyle treatment including at least four visits per year. Pharmacotherapy is emerging as a new treatment option, and bariatric surgery is offered to 30–50 Swedish adolescents per year. Obesity treatment should be offered by paediatric health care professionals in multidisciplinary teams and is free of cost for all children and adolescents in Sweden. Lifestyle treatment for childhood obesity has been offered for more than 15 years in Swedish outpatient clinics. The effectiveness of this treatment has not improved during these years, and treatment outcomes have even deteriorated [17]. In addition, high dropout rates from lifestyle treatment have been reported from the Swedish Childhood Obesity Treatment Register (BORIS).

Dietary interventions that reach further than solely counselling are scarce within the treatment of childhood obesity. The present study explored weather involving the whole family in a practical and participatory intervention, cooking a large variety of recipes, might be a novel treatment option. The aim of this study was to evaluate if Family Meals on Prescription (FMP), using a subsidized weekly grocery bag, in combination with lifestyle treatment, was tolerable and could improve weight status and metabolic parameters in children with overweight or obesity and thereby provide a novel treatment option.

## Methods

### Settings

The study was a prospective randomized controlled trial conducted from January 2019 through January 2022 at the two Paediatric Obesity Clinics in Kungsbacka and Varberg, Sweden. The trial was conducted in accordance with the principles of the Declaration of Helsinki and was approved by the regional ethical review board in Gothenburg (no 2018/636). The study has been registered in ClinicalTrials.gov (NCT05225350). Written informed consent was obtained from all parents and/or legal guardians as well as from the adolescents who were 15 years old at inclusion.

### Participants and recruitment

The inclusion criteria were children and adolescents between 5 and 15 years of age receiving lifestyle treatment for obesity. Eligible patients were invited to an information meeting and were informed in connection with visits at the clinic. The only exclusion criterion was insufficient knowledge of the Swedish language. The company responsible for the grocery bags was able to deliver a maximum of 50 grocery bags every week for 3 months. With the 1:1 randomization, the maximum study population was therefore 100 families.

### Randomization

Participants were randomized in a 1:1 ratio at each site through sealed envelope randomization, prepared for 100 patients, to the intervention (FMP in addition to lifestyle treatment) or control group (lifestyle treatment alone).

### Intervention

All participants received lifestyle treatment before, during and after the intervention. The intervention may be seen as an add-on treatment for families receiving lifestyle treatment. The intervention was to receive a subsidized bag containing groceries and pedagogical recipes for five family dinner meals per week during a period of 3 months for the entire family. The recipes were designed by a dietitian at the company providing the bags and were to be in line with the Swedish Food agency’s recommendations [18]. During the intervention, different sources of protein were divided throughout the meals, and there was always at least 1 day of meat, fish as well as a vegetarian day per week. The prepacked grocery bags were obtained from a local store. The standard price of the bag was 619 SEK (53 USD) per week, but to ensure that all families could participate regardless of economic status, the price was reduced to 133 SEK (11 USD) per week through a subsidization from Gen Pep Foundation. The start of the intervention was divided into spring and autumn. To ensure a controlled portion size, all participants in the study were given an age-adjusted Meal Sizer® (a portion control tool) that could help with portion size and emphasized the goal of one serving per meal (supplement 1).

All study participants received lifestyle treatment consisting of counselling concerning healthy nutrition, physical activity, the importance of sleep and stress management throughout the whole trial and follow up period. The health professionals were paediatric nurses, dietitians and paediatricians with specific training in the field of childhood obesity. The treatment was directed toward the caregivers and with increasing age to the child. According to national guidelines, four visits per year to the obesity team and yearly laboratory and physical assessments were set as a minimum at the start of the trial; however, the treatment was individualized based on the wishes and needs of the patient. The dietary advice included counselling regarding the amount of fruits and vegetables, meal patterns and the importance of avoiding ultra-processed foods such as desserts, sugar-sweetened beverages and deep-fried foods. The level of physical activity was evaluated, and individualized advice was given. Municipal support was offered for children with a highly sedentary lifestyle. During the intervention period, patients in both groups were asked to report any major changes that might affect BMI development such as illness or changes in medication.

### Blinding

The data was collected and analysed by an investigator who was blinded to the intervention.

Due to the nature of the intervention, neither participants nor members of the obesity team were blinded to the intervention.

### Outcomes

The primary outcome of the present study was change in BMIz. Weight and height were measured at in-person visits at the clinic. Participants were weighed on electronic step scales, and a wall-mounted stadiometer was used to measure height. BMI was calculated as weight (kg) divided by height squared (meters), and BMIz was obtained from Swedish national growth standards [19]. We calculated individual change in BMIz at 3 and 12, 18–24 months after the start of the intervention (ΔBMIz). All caregivers and participants responded to a written question regarding how often the family ate dinner together at baseline, 3 months and 12 months. The response options were 0, 1–2, 3–4, 5–6 or 7 times per week. Data regarding duration of treatment, number of health care contacts and treatment with central stimulants known to cause weight loss as well as laboratory results concerning metabolic biomarkers for fasting glucose, fasting insulin, HbA1c, blood lipids and liver markers were retrieved from medical charts. Data regarding how families collect the grocery bags was obtained from the grocery store.

### Statistical analysis

Baseline characteristics of the study population are presented as means and standard deviations for continuous variables and as number and percentages for categorical variables. Normal distribution of the continuous variables was assessed through plotted histograms. The number of contacts and duration of lifestyle treatment before the start of intervention are presented using median and range due to non-normal distribution. Student’s *t* test was used to compare means at baseline as well as at follow up between the intervention and control groups for numerous variables such as age, blood samples, BMIz and change in BMIz, and a Mann–Whitney *U* test was used to compare median for time in treatment before study and number of visits. Data was analysed per protocol; no imputation was performed for missing values. In addition, an intention to treat analysis was performed for the 3-month finding regarding BMIz. In the analysis, the participants were analysed in accordance with their original group allocation at study start. Furthermore, we used a Chi-square test to compare the distribution of sex and weight status severity (overweight or obesity). To account for baseline BMI, we performed an analysis of covariance with adjustment for BMIz at baseline. To investigate how the habit of eating together was affected, the Friedman test was performed. A *p* value of ≤ 0.05 was considered significant.

The IBM SPSS Statistics (version 28.0.1.1) was used for the statistical analysis.

### Sensitivity analyses

Sensitivity analyses were performed to test possible confounders such as excluding participants who started treatment with central stimulant for ADHD during the intervention. An analysis was also performed to investigate the effects of the siblings who changed groups after randomization. Moreover, we also analysed study outcomes with the study participants divided on the median number of contacts with the clinic and on spring or autumn start of intervention since the COVID-19 pandemic started before autumn starters had their 12-month follow up.

## Results

**Fig. 1:**
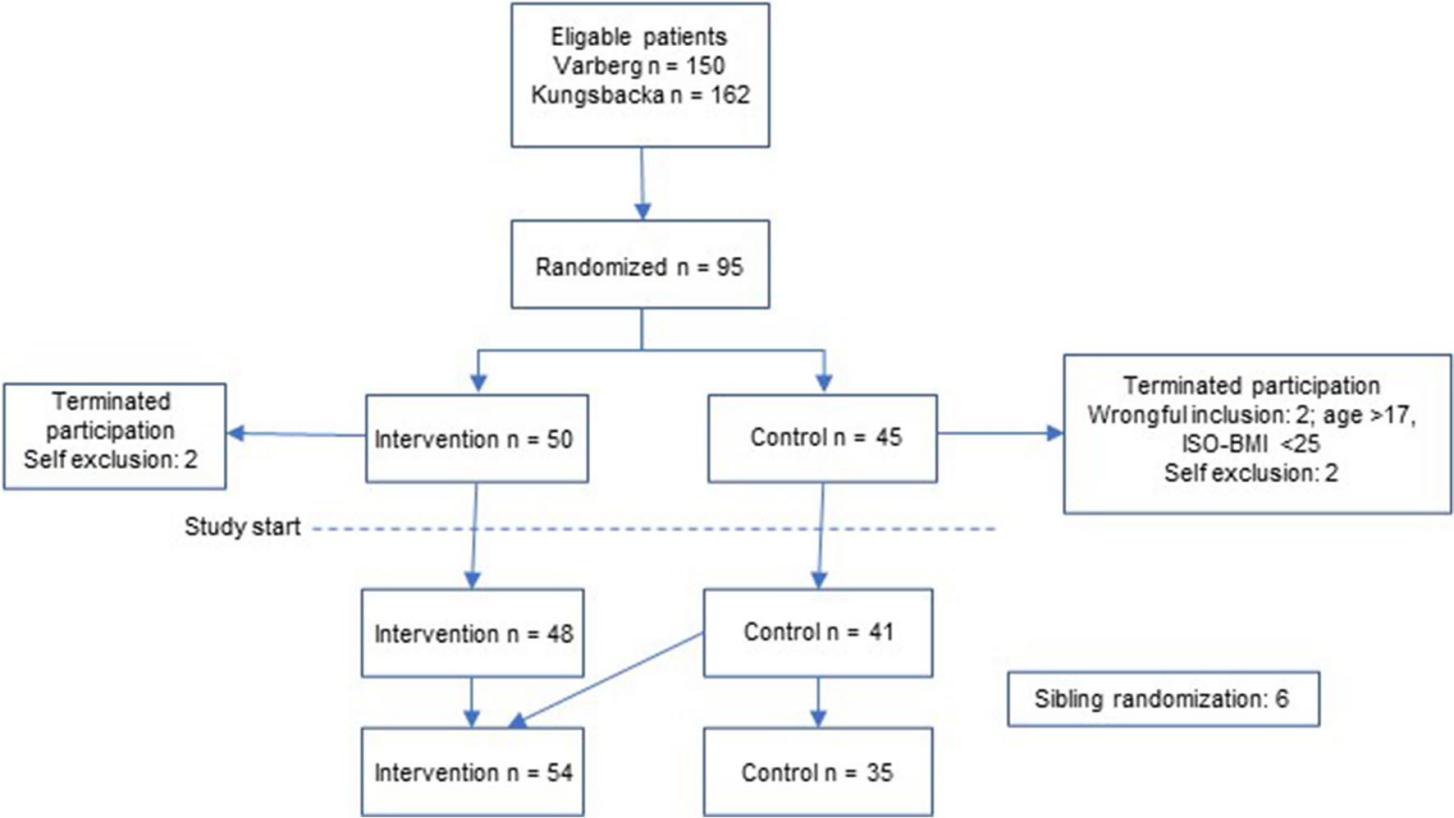
Participant flow chart

**Fig. 2:**
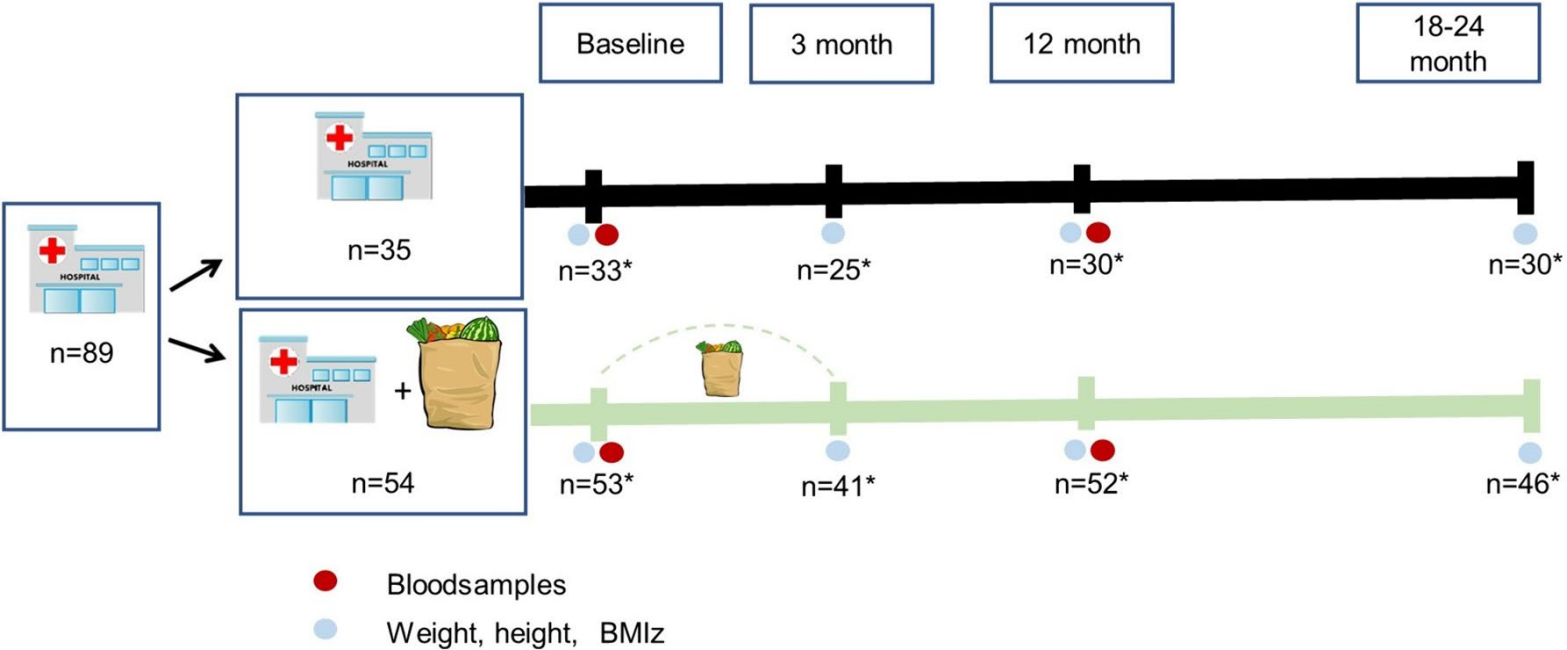
Study design. *For ∆BMIz. Dark line represents lifestyle treatment alone, light line represents lifestyle treatment plus Family Meals on Prescription during the first 3 months

There were 312 patients eligible for inclusion in this study. All were invited to attend an information meeting; 95 chose to be included. After randomization, four patients withdrew consent, and two patients were excluded due to wrongful inclusion. In total, 89 patients, 43 boys and 46 girls, entered the study. Since some of the included patients were from the same family and were randomized individually to different groups, a decision was made to allow these included families to choose group based on their preference (*n* = 6). After these changes, the intervention group consisted of 54 participants, and the control group was composed of 35 individuals (Fig. 1). The retention rate was 92% 12 months after inclusion and 85% 18–24 months after inclusion (Fig. 2).

There were no significant differences between the groups in baseline data. Gender distribution and rates of obesity and overweight were essentially similar. The mean age at inclusion was 10.3 (± 2.7) in the intervention group and 11.0 (± 2.6) in the control group (*p* ≥ 0.05). The mean BMIz at baseline was 3.0 (1.48–5.54) in the intervention group and 2.9 (1.68–4.63) in the control group, respectively (*p* > 0.05) (Table 1).

**Table 1:** Characteristics of the participants at baseline. The data is presented as mean ± standard deviation. For sex, overweight/obesity and use of stimulant distribution and percentage were used. The number of months in treatment prior to study start is presented as median, minimum and maximum

	Family Meals on Prescription (*n* = 54)	Control group (*n* = 35)	*P* value
Female sex, *n* (%)	27 (50)	19 (54)	0.69
Age, year, mean ± SD (*n*)	10.3 ± 2.7 (54)	11.0 ± 2.6 (35)	0.18
Overweight/Obesity *n* (%)	12 (23)/41 (77)	6 (18)/27 (82)	0.62
BMIz, mean ± SD (*n*)	3.0 ± 0.8 (53)	2.9 ± 0.8 (33)	0.51
Number of months in treatment prior to study start, median; min, max	18.5; 0, 63	20.0; 0, 81	0.45
HbA1C, mmol/mol mean ± SD (n)	35.2 ± 3.0 (38)	34.4 ± 4.0 (31)	0.37
Fasting plasma glucose, mmol/L, mean ± SD (*n*)	5.1 ± 0.4 (40)	5.1 ± 0.3 (29)	0.80
Insulin, mU/L, mean ± SD (*n*)	18.6 ± 14.3 (38)	17.8 ± 12.7 (29)	0.82
Cholesterol, mmol/L, mean ± SD (*n*)	4.2 ± 0.8 (39)	4.2 ± 0.5 (31)	0.59
High-density lipoprotein, mmol/L, mean ± SD (*n*)	1.3 ± 0.2 (39)	1.3 ± 0.2 (31)	0.41
Low-density lipoprotein, mmol/L, mean ± SD (*n*)	2.7 ± 0.8 (39)	2.7 ± 0.5 (30)	0.67
Triglycerides—mmol/L, mean ± SD (*n*)	1.0 ± 0.5 (39)	1.1 ± 0.5 (30)	0.47
ALAT, µkat/L, mean ± SD (*n*)	0.4 ± 0.2 (40)	0.5 ± 0.4 (30)	0.39
25 OH-vitamin D, nmol/L, mean ± SD (*n*)	64.7 ± 20.0 (36)	58.8 ± 14.3 (28)	0.17
Using central stimulants, *n* (%)	15 (8)	14 (5)	0.95

**Fig. 3:**
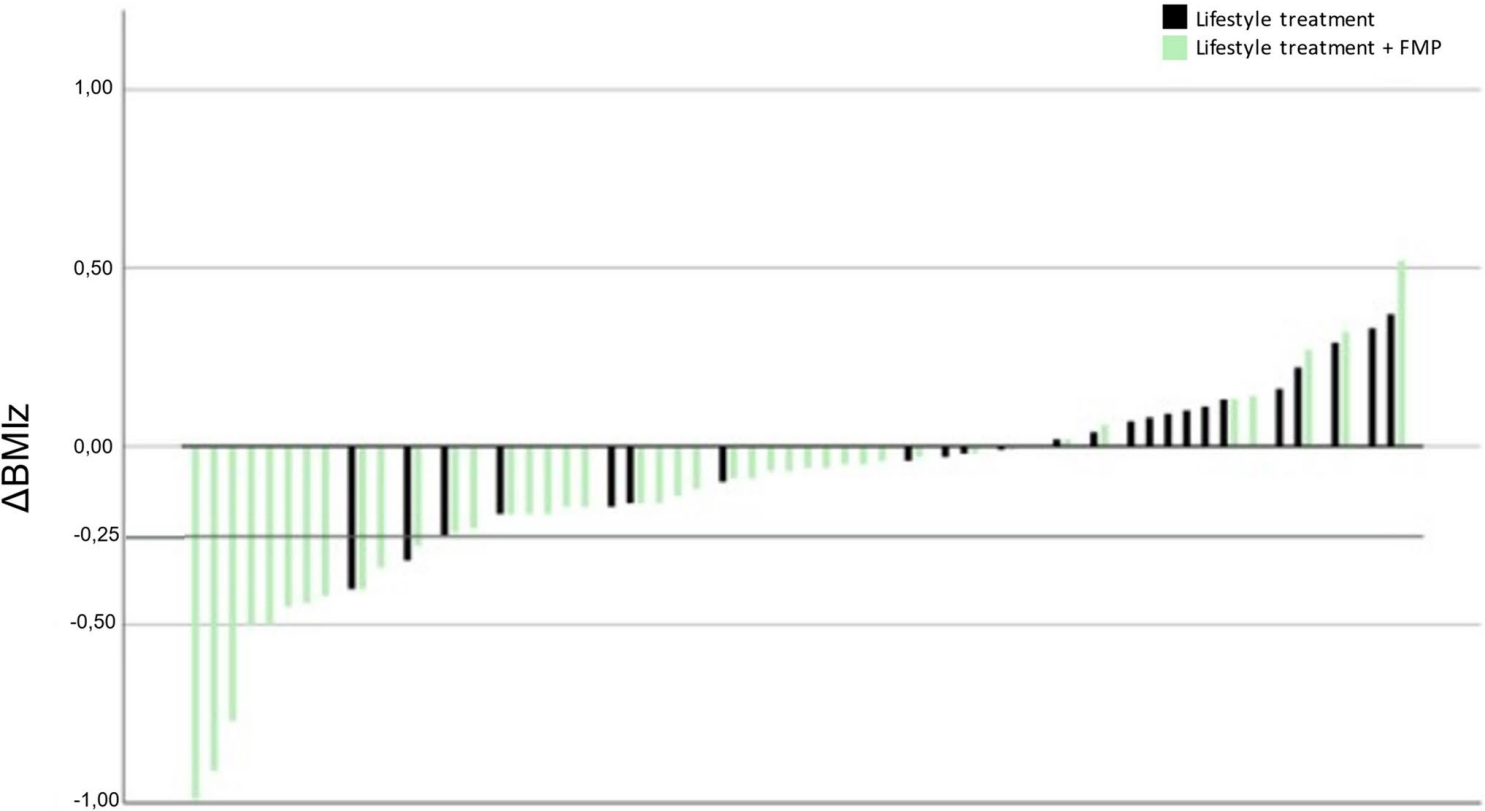
Waterfall plot describing change in BMIz from baseline to 3 months follow up. Dark bars represent lifestyle treatment alone and light bars represent lifestyle treatment plus Family Meals on Prescription (FMP)

The intervention FMP was well tolerated by all participants and their families. Compliance to collecting the bag from the grocery store throughout the intervention was 100%. The mean BMIz reduction at 3 months was significantly greater in the intervention group compared to the control group (− 0.17 SDS ± 0.29 vs + 0.01 SDS ± 0.19, *p* < 0.01). There were significantly more participants with a reduced BMIz in the intervention group compared to the control group (83%, 34/41 vs 44% 11/25, *p* < 0.01). During the 3-month intervention, a total of 13 children had a change in BMIz greater than − 0.25, 11 (20%) of these belonged to the intervention group, compared with 2 (5%) in the control group (*p* > 0.05) (Fig. 3). When an intention to treat analysis was performed the intervention group had a greater reduction in BMIz at 3 months compared to the control group (− 0.16 ± 0.28 vs − 0.04 ± 0.0.26, *p* > 0.05); however, the difference fell just short of significance.

The mean BMIz change after 12 months compared to baseline was smaller in the intervention group compared to the control group (− 0.03 SDS ± 0.47 vs − 0.33 SDS ± 0.64; *p* < 0.05). The differences from baseline increased further after 18–24 months when the control group showed a decreased BMIz of − 0.44 BMIz, and the intervention group had an increase of 0.03 (*p* < 0.01) (Table 2). There were no differences between the groups regarding biomarkers, except for a significantly higher HbA1c in the control group, compared with the intervention group at the 12-month follow up (Table 3). The number of contacts from start to 12 months follow up (median and interquartile range (IQR)) was 4 (IQR 2–5) for the intervention group and 4 (IQR 3–5) for the control group. Between 12 and 18–24-month follow-up, the median number of contacts was 1 (IQR 1–2). There was a median of six contacts with the clinic during the whole study period (0–24 months).

**Table 2:** Mean change in BMIz from baseline to 3-, 12- and 18–24-month follow-up. The difference between groups is presented and data given as means ± standard deviation

	Baseline to 3 months			Baseline to 12 months			Baseline to 18–24 months		
End point	Family Meals on Prescription	Control group	*P* value	Family Meals on Prescription	Control group	*P* value	Family Meals on Prescription	Control group	*P* value
Delta BMIz (*n*)	− 0.17 ± 0.29 (41)	0.01 ± 0.19 (25)	< 0.01	− 0.03 ± 0.47 (52)	− 0.33 ± 0.64 (30)	0.03^a^	0.03 ± 0.55 (46)	− 0.44 ± 0.77 (30)	< 0.01

^a^Significant difference did not stand after participants who started treatment with central stimulants were excluded

**Table 3:** Mean change in blood samples from baseline to 12-month follow-up. The difference between groups is presented and data given as means ± standard deviation

	Change from baseline to 12 months		
End point	Family Meals on Prescription	Control group	*P* value
HbA1C—mmol/mol (*n*)	0.60 ± 1.68 (35)	1.79 ± 1.82 (24)	0.01*
Fasting plasma glucose—mmol/L (*n*)	− 0.02 ± 0.39 (37)	− 0.14 ± 0.47 (22)	0.30
Insulin—mU/L (*n*)	1.63 ± 11.98 (35)	0.45 ± 9.74 (22)	0.70
Cholesterol—mmol/L (*n*)	− 0.23 ± 0.59 (36)	− 0.20 ± 0.53 (24)	0.81
High-density lipoprotein—mmol/L (*n*)	0.04 ± 0.19 (36)	0.04 ± 0.19 (24)	1.00
Low-density lipoprotein—mmol/L (*n*)	− 0.25 ± 0.48 (36)	− 0.24 ± 0.44 (23)	0.88
Triglycerides—mmol/L (*n*)	− 0.06 ± 0.47 (36)	− 0.17 ± 0.56 (23)	0.44
ALAT—µkat/L (*n*)	− 0.3 ± 0.20 (37)	− 0.13 ± 0.37 (23)	0.17
25 OH-vitamin D—nmol/L (*n*)	− 2.88 ± 17.86 (33)	− 2.90 ± 15 (20)	0.10

^*^Significant change

Sensitivity analyses for the primary endpoint were performed without siblings who changed groups after randomization, individuals who initiated central stimulant treatment for ADHD during the trial and according to number of contacts with the clinic during the trial and study start in spring or autumn. When the six siblings who changed group after randomization were excluded from analysis, the results were still maintained. Exclusion of participants who started stimulant treatment (*n* = 3) did not affect the significance of change in BMIz at the 3-month follow up. At the 12-month follow-up, five participants in the control group had started stimulants. After exclusion of these participants, there was no longer a significant difference between the groups concerning BMIz at the 12-month follow-up. Two participants in the intervention group and one in the control group started stimulants before the 18–24-month follow-up. The sensitivity analysis without these individuals did not affect the results. The participants were divided in to two groups, less than six contacts (*n* = 41) and six contacts or more (*n* = 48), respectively. Participants with less than six contacts had a greater decrease in change in BMIz at 18–24 months. Moreover, time of study start did not affect any results at any time points. Similar results regarding BMIz over time were seen when using an analysis of covariance adjusting for BMIz at baseline at 3 months (2.8 ± 0.90 vs 3.0 ± 0,97, *p* < 0.01), 12 months (2.97 ± 0.97 vs 2.55 ± 0.92, *p* = 0.02) and 18–24 months (3.07 ± 1.04 vs 2.52 ± 0.99, *p* < 0.01).

When the content of the grocery bags was double checked after the intervention took place, the average content of one meal was 659 kcal, 39 E% carbohydrates, 21 E% protein and 40 E% fat (17 E% saturated fat). The average amount of fibre was 2.7 g/MJ, and vegetable was 138 g/meal. The participants answered the question in a questionnaire “How often does the family sit down and eat dinner together?” Twenty-nine participants in the intervention group and 10 in the control group responded to the questionnaire at all three time points. The changes in frequency of family meals were evaluated after the 3-month intervention. Participants in the intervention group (*n* = 40) and in the control group (*n* = 14) responded (total response rate of 61%). An increase in the number of shared family meals per week was observed in the intervention group compared to baseline; however, the results fell just short of statistical significance.

## Discussion

In this trial, children and adolescents with overweight or obesity were randomized to a 3-month intervention including Family Meals on Prescription delivered as a subsidized grocery bag, in combination with lifestyle treatment, or lifestyle treatment alone. The group receiving the dietary intervention had a significantly greater reduction in BMIz compared to the group receiving lifestyle treatment alone after the 3-month-long intervention. In addition, a greater number of patients in the intervention group than the control group achieved a clinically relevant reduction in BMIz. However, these results were not sustained throughout the follow up period; on the contrary, the control group had a greater reduction in BMIz after 18–24 months. The families in the intervention group received a grocery bag containing a wide range of different pedagogical recipes. The fact that all families in the intervention group continued with the grocery bag throughout the intervention period indicates that the intervention was well tolerated. The results from FMP in the present study are in accordance with two recently published studies regarding a dietary intervention using vouchers for purchasing fruits and vegetables [20, 21]. The “Produce on Prescription-study” demonstrated significant improvement in dietary patterns among children and adults; however, in that study, only the adults had significant changes in BMI. Of note, the “Fruit and Vegetable Prescription Program” showed significant reduction in BMI in children with obesity; however, the study did not have a control group [21]. The scarcity in randomized controlled trials investigating dietary treatment interventions with a participatory approach given in the home setting highlights the need for further prospective randomized and controlled trials aimed specifically for children and adolescents living with obesity [9, 20].

There was a heterogeneous treatment response in terms of change in BMI in both groups in the present study. This is in accordance with many studies in the field of childhood obesity [22–24]. This heterogeneous response emphasizes the need for personalized medicine in the treatment of paediatric obesity, i.e. to identify and characterize patients who would benefit the most from future treatment involving an intensive dietary intervention such as the one in the present study. The reduction in BMIz may be explained by the families improving their mealtime routines as well as altering the quality and quantity of food since the meals were composed of a variety of recipes. These factors may lead to a decreased calorie intake and have been positively associated with healthier diet-related outcomes such as BMI in previous studies [13–15]. Further, a trend was shown indicating that the intervention group had more frequent family meals after the intervention. These results are of importance since significant associations have been shown between higher family meal frequency and a lower BMI, more healthy diet and better overall diet quality as well as less unhealthy diet [13]. Of note, the intervention was well tolerated suggesting this intervention could be offered to many families in need of a dietary alteration.

Weight regain after a successful dietary or pharmacological intervention is frequently observed in studies concerning childhood obesity and is also shown in the present study where the effect of the 3-month intervention on BMIz was not sustained at the 12–24-month follow up. This supports the notion that obesity is a chronic disease that requires continuous treatment and that a longer intervention may be more effective in the long term [22–25].

As pharmacotherapy was not an option when this trial was conducted, none of the patients received GLP-1 analogues. At present, when pharmacotherapy is offered, the combination with FMP may show additive effects and should be investigated in future studies. However, the participants in this trial had a mean age of 10.6 and 11 years, respectively, and for many of the study participants, pharmacotherapy or Metabolic Bariatric Surgery would not have been offered in a clinical setting at present date.

A large number of factors may have affected the study results regarding change in BMIz. Previous studies have shown that the age at treatment start, the obesity severity and number of contacts with health care are of importance for treatment outcomes [26–28]. In this trial, there were no significant differences between the groups concerning these factors at baseline that may explain the difference seen in BMIz development. Moreover, Swedish studies have demonstrated a high level of neurodevelopmental disorders among children with obesity, and treatment with stimulants has been shown to decrease BMI in up to 50% of children receiving stimulants [29, 30]. In a sensitivity analysis excluding individuals who started treatment with stimulants, the significant difference in BMIz between the groups at 12 months was not maintained. This finding indicates that treatment with stimulants may, at least partly, explain the results seen at 12 months, further underlining the need to identify and treat neurodevelopmental comorbidities in paediatric obesity. In addition, the pandemic had a profound effect on the entire society but also on the obesity treatment during the follow up period since it affected the accessibility to regular visits. During the intervention period, the lifestyle treatment was unaffected; however, the pandemic significantly decreased the number of visits to the clinic during the 12–24-month follow up period rendering it difficult to identify the reasons behind the greater reduction in BMIz in the control group. There is previous evidence that the efficacy of obesity treatment was affected by the pandemic [31, 32]. This might also explain the heterogeneous response demonstrated after the 12–24 months of follow up period and may be one of many explanations regarding the greater reduction in BMIz in the control group after 18–24 months.

### Strengths and limitations

A strength of this study is that it was a prospective and randomized trial where the intervention was added to the real-life care while there are few randomized controlled trials investigating dietary interventions with a participatory approach delivered in the home setting in children living with obesity. Another strength is the long follow-up period and the high retention rate. In the present study, 92% of patients remained in the study after 12 months in contrast to the 40% loss to follow up that has been reported from routine treatment for paediatric obesity from the Swedish quality register, BORIS. Furthermore, the intervention was not solely directed toward the child in treatment. Instead, it involved the whole family in the changes at mealtime, and it took place in the home environment. These two factors have been previously reported to be of importance in the treatment of childhood obesity [4, 10–12, 16].

There are some limitations in the study. Simple size calculation was not performed on the basis of the primary endpoint but on the maximum of bags that could be distributed. This is a limiting factor. The randomization was done individually so that children from the same family could end up in the different groups initially. After adjusting so that siblings were put in the same group, it consequently affected the group sizes. This was not anticipated when the study was designed. However, sensitivity analysis showed that this did not affect the results. When the content of the bags was double checked, the result showed a higher amount of fat and saturated fat than recommended levels. A more optimized grocery bag may contribute to improve future results; however, this needs additional investigation. Another limitation is the lack of information concerning the frequency of shared family meals and general food consumption as well as information regarding the socioeconomic status and the level of physical activity.

## Conclusion

The intervention FMP was well tolerated, and all families completed the whole intervention. FMP in combination with lifestyle treatment led to a significantly greater reduction in BMIz than lifestyle treatment alone after the 3-month intervention. These findings are in line with research showing a significant relationship between frequent family meals and better nutritional health in younger and older children. Further studies are needed to evaluate if a longer intervention with FMP may have a more long-standing effect, since the results did not stand throughout the follow-up period. The results are important since lifestyle treatment is at present the only treatment alternative for many of the patients treated in outpatient clinics.

## Role of funder

The subsidized price for the grocery bag was funded by the foundation Generation Pep (www.generationpep.se). Research time was funded through Agreement for Medical Education and Research, ALF, and the Halland Regional Research board (Halland 907,371).

## Supplementary Information

Below is the link to the electronic supplementary material.

Supplementary file1 (DOCX 1166 KB)

## Data Availability

No datasets were generated or analysed during the current study.

## Abbreviations

BMI: Body mass index
BMIz: Body mass index z-score
FMP: Family Meals on Prescription
SEK: Swedish Krona
USD: US Dollar
GLP-1: Glucagon-like peptide 1
