# Supplementary material for: Family meals on prescription as treatment for childhood obesity—a randomized controlled trial
Source: Eur J Pediatr. 2024 Sep 9;183(11):4857–66. doi: 10.1007/s00431-024-05744-8 (PMC11473609; doi:10.1007/s00431-024-05744-8)

**Supplement 1**

The Mealsizer® meal measure-volumes are calculated by licensed dietitian, to provide the right amount of energy and nutrition for a meal, based on Nordic Nutrition Recommendations (NNR) (also correlated to international guidelines, as the DGE and WHO's nutritional recommendations). The calculations are based on a normally active energy consumer (children from approx. 6-12 years 1500 kcal/day, adolescents 12-18 years 2100 kcal/day).


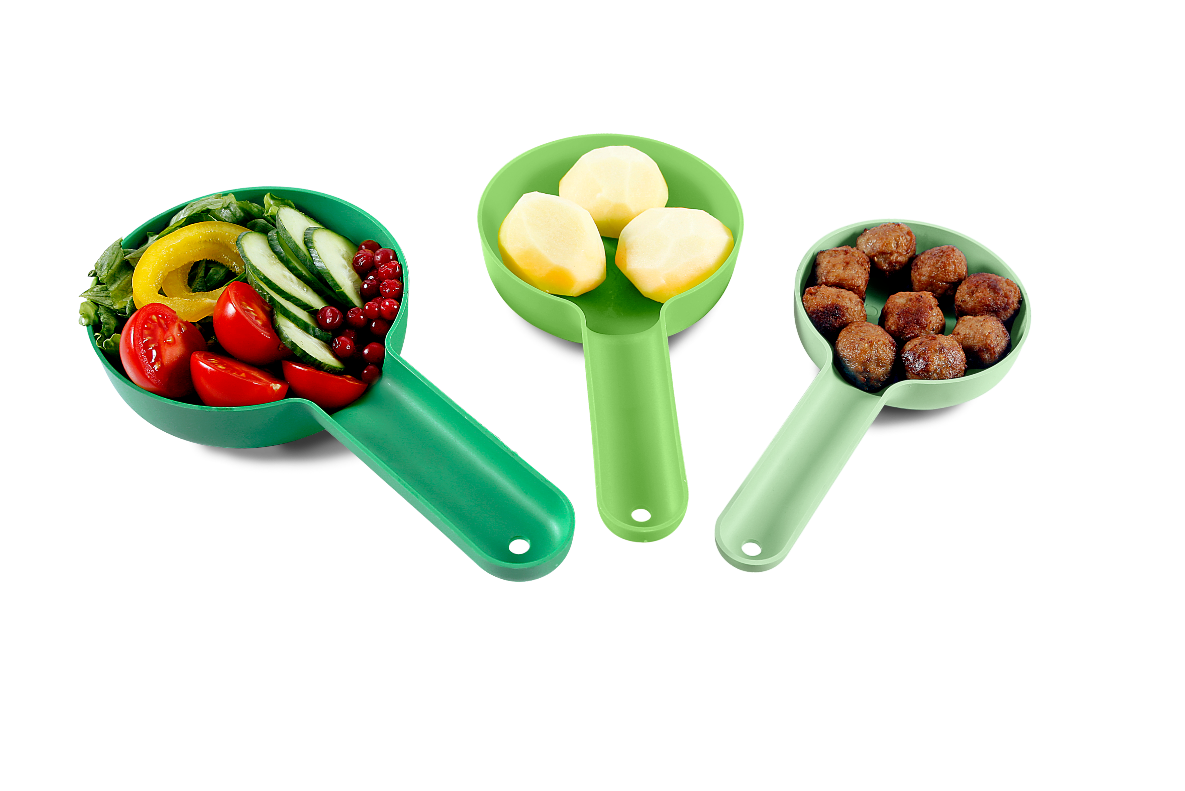

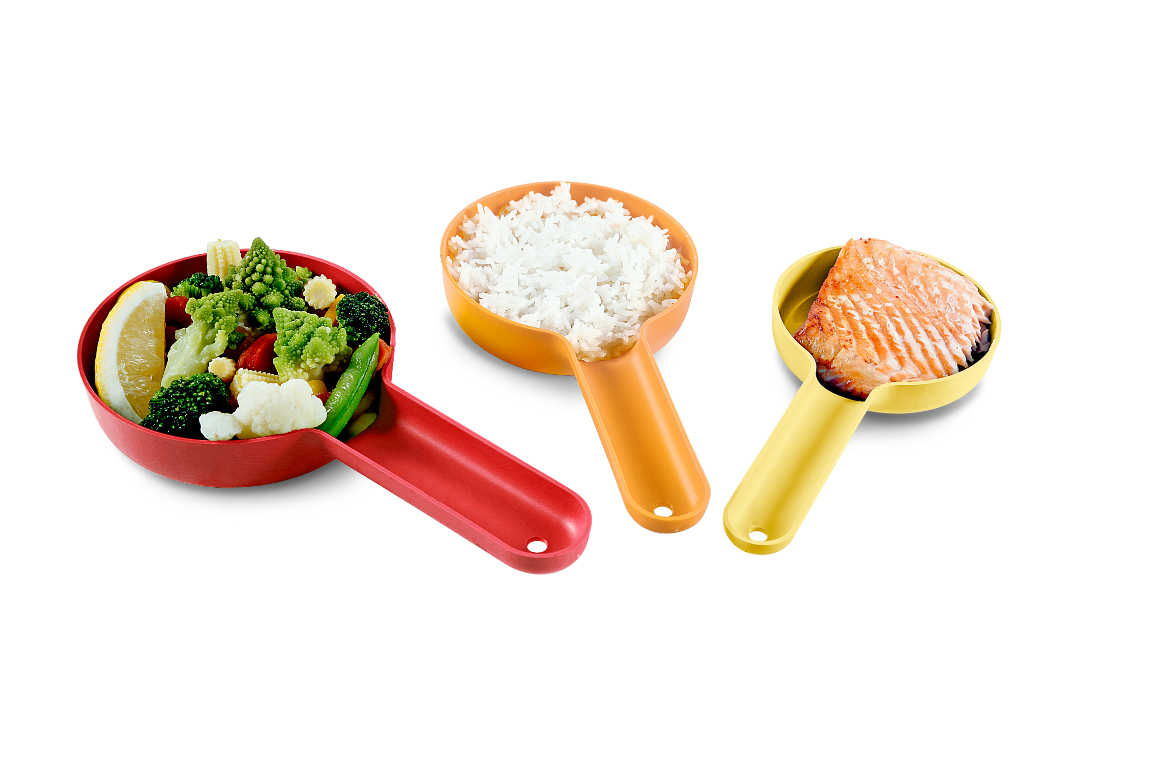

Supplement: Supplementary file 1 — Supplementary file1 (DOCX 1166 KB) [file 431_2024_5744_MOESM1_ESM.docx]
